# A Personalised 3D-Printed Dental Plaque Removal Mouthguard for Older Adults

**DOI:** 10.1016/j.identj.2023.04.005

**Published:** 2023-05-16

**Authors:** Hao Ding, Meng Zhang, Brian Lo, Karfield K.F. Chan, Edward C.M. Lo, James K.H. Tsoi

**Affiliations:** a Dental Materials Science, Division of Applied Oral Sciences & Community Dental Care, Faculty of Dentistry, The University of Hong Kong, Pokfulam, Hong Kong; b Dental Public Health, Division of Applied Oral Sciences & Community Dental Care, Faculty of Dentistry, The University of Hong Kong, Pokfulam, Hong Kong

**Keywords:** 3D printing, Mouthguard, Dental plaque removal, TMQHPI, Older adult

## Abstract

**Objectives:**

The aim of the present study was to examine the plaque removal effectiveness of a personalised 3D-printed dental plaque removal mouthguard device in a clinical trial setting.

**Methods:**

A personalised 3D-printed mouthguard was developed to clean dental plaque using micro-mist. A clinical trial was conducted to examine the plaque removal effectiveness of this device. The clinical trial recruited 55 participants (21 males and 34 females) with an average age of 68.4 years (range, 60–81 years). Dental plaque was dyed by plaque disclosing liquid (Ci). Turesky Modification of the Quigley-Hein Plaque Index (TMQHPI) was used to evaluate the level and rate of plaque formation on the tooth surface. The TMQHPI was recorded and intraoral photos were taken before and after mouthguard cleaning. The plaque removal rate was calculated based on TMQHPI and intraoral photos (pixel-based method) before and after cleaning.

**Results:**

The personalised 3D-printed micro-mist injection mouthguard can be effective in dental plaque removal on tooth and gingiva, and the effectiveness lies between that of a manual toothbrush and a mouth rinse. The newly proposed pixel-based method can be a practical, high sensitive tool to evaluate the level of plaque formation.

**Conclusions:**

Under the conditions of the present study, we conclude that the personalised 3D-printed micro-mist injection mouthguard can be useful in reducing dental plaque and may be especially suitable for older adults and disabled people.

## Introduction

Older adults are at great risk for dental caries and periodontal diseases due to the accumulation of dental plaque in the oral cavity.^1^ The high prevalence of xerostomia amongst older adults could increase the rate of microbial colonisation,^2^ whilst decreasing hand function due to age, trauma, or disease^3^^,^^4^ makes it more difficult to clean the teeth thoroughly. Timely dental plaque removal is essential to minimise surrounding inflammation^5^ and avoid the calcification of plaque attaching to the tooth.^6^ Hence, assisting older adults to remove dental plaque in a timely manner is beneficial to their oral health and even general health.

Hong Kong is an aging society, with more than 21% of its citizens expected to be older than 65 years by 2024.^7^ However, due to staff shortages and inadequate funding,^8^ both institutional and home care services struggle to provide sufficient oral care for older adults who have difficulties with self-care. Consequently, the oral health status of older adults, particularly those in long-term care, is far from satisfactory.^9^ A 2011 oral health survey^10^ revealed that more than 50% of institutionalised older adults had untreated dental caries, with an average of 2.2 to 3 decayed teeth per person. Furthermore, almost all institutionalised older adults had periodontal problems, such as gum bleeding and attachment loss of more than 4 mm.

Various mechanisms are used for tooth cleaning, including mechanical abrasion with a toothbrush, water pressure using an oral irrigator, and antiseptic ingredients found in mouth rinse. Another method, micro-mist, combines water droplets and air flow to create high-velocity steam air micro-mists. Theoretically, micro-mist has greater teeth-cleaning ability compared with water droplets or air flow alone due to the higher kinetic energy of the water droplets in micro-mist form.^11^

Hihara et al^12^ developed a dental plaque removal device utilising micro-mist spray, and it was effective both in vitro^12^ and in vivo.^13^ However, it is a handheld device with single outlet and requires an operator to handle. In this study, a personalised 3D-printed micro-mist injection mouthguard was invented. The invention has certain special design features that aim at helping older adults and disabled people to clean their oral cavities safely and effectively even by themselves. The mouthguard was made according to older adults’ personal dental arch form, whereas specially designed outlets focusing on gum margin on each tooth were embedded and printed with soft polymeric material. By connecting the mouthguard to air and water channels, a minimal amount of water forms micro-scale mists at the outlets directly striking on the dental plaques at the gumline. This study aimed to verify the plaque removal effectiveness of this device in a human trial.

## Materials and methods

### Mouthguard fabrication

**Fig. 1:**
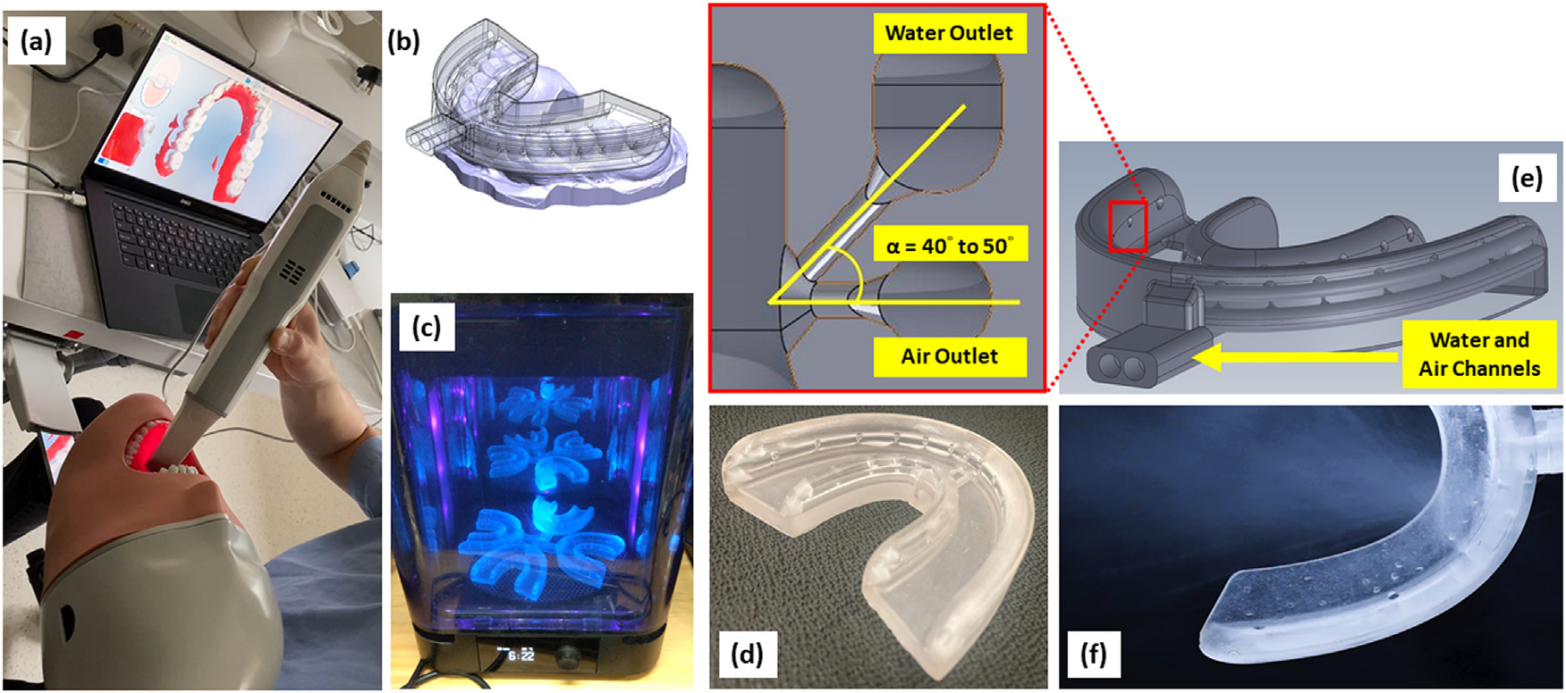
Schematic diagram of the personalised mouthguard: A, impression taking; B, design adjustment according to the digital impression; C, SLA 3D printing; D, a prototype of the mouthguard; E, close-up view of air and water nozzle design; F, close-up view of micro-mist generation.

As shown in Figure 1, the personalised mouthguard was produced by utilising patients’ digital dental impressions scanned by an intraoral scanner (iTero, Align Technology Inc.). The digital impression was then merged with the mouthguard design in CAD software (SolidWorks, Dassault Systémes SolidWorks Corp.), and the shape and outlet locations of the mouthguard were adjusted according to the patient's digital impression. The personalised mouthguard was then 3D-printed using a stereolithography (SLA) printer (Form 3, Formlabs) with elastomeric resin (95 wt% acrylate monomer, 5 wt% urethane dimethacrylate). The micro-mist was generated at the outlets of the mouthguard by mixing the air and water coming through 2 separated channels that connected to a dedicated machine with specific water and air pumps. The intersection angle (α) between air and water nozzles was designed between 40° and 50° (Figure 1E), with a water flow rate of 25 to 85 mL/min and an air flow rate of 30 to 50 L/min.

### Clinical trial

#### Participants

A clinical trial was conducted from September 2021 to September 2022 to evaluate the effectiveness of the mouthguard device in removing dental plaque. To be eligible for the trial, participants had to be aged 60 or older, have all 6 anterior teeth in the first and third quadrants (teeth 11–13 and 31–33), and be able to read or speak. Patients with oral mucosal diseases including those undergoing treatment or recovery were excluded from the study. After screening, 55 participants (21 males and 34 females) with an average age of 68.4 years (range 60–81 years) were included in the trial. All participants provided informed consent and signed the consent form approved by the University of Hong Kong (HKU) and the Hospital Authority Hong Kong West Cluster (HA HKW) (Ref No: UW 21-259).

#### Study design and procedures

A custom-made mouthguard was created for each participant. The participants were instructed not to brush their teeth within 8 hours before the trial. During the trial, participants rinsed the mouth with water to remove debris, plaque-disclosing liquid (Ci) was subsequently applied to dye the plaque, and then power-rinsing was applied to remove extra staining. The teeth in the first and third quadrants were examined and scored based on Turesky Modification of the Quigley-Hein Plaque Index (TMQHPI) before and after the application of the personalised mouthguard. Photos of dyed anterior teeth (11–13 and 31–33) were taken using an iPhone, and 6 photos from different angles were taken for each participant to ensure that the buccal surface of each tooth can be properly visualised. Next, the custom-made mouthguard was used to clean the teeth, and water and air power were adjusted according to the participants’ remaining teeth number. The TMQHPI was examined and photos were taken again following the aforementioned procedures. Finally, residual dyes were rinsed after all the procedures.

### Evaluation of plaque removal effectiveness

**Fig. 2:**
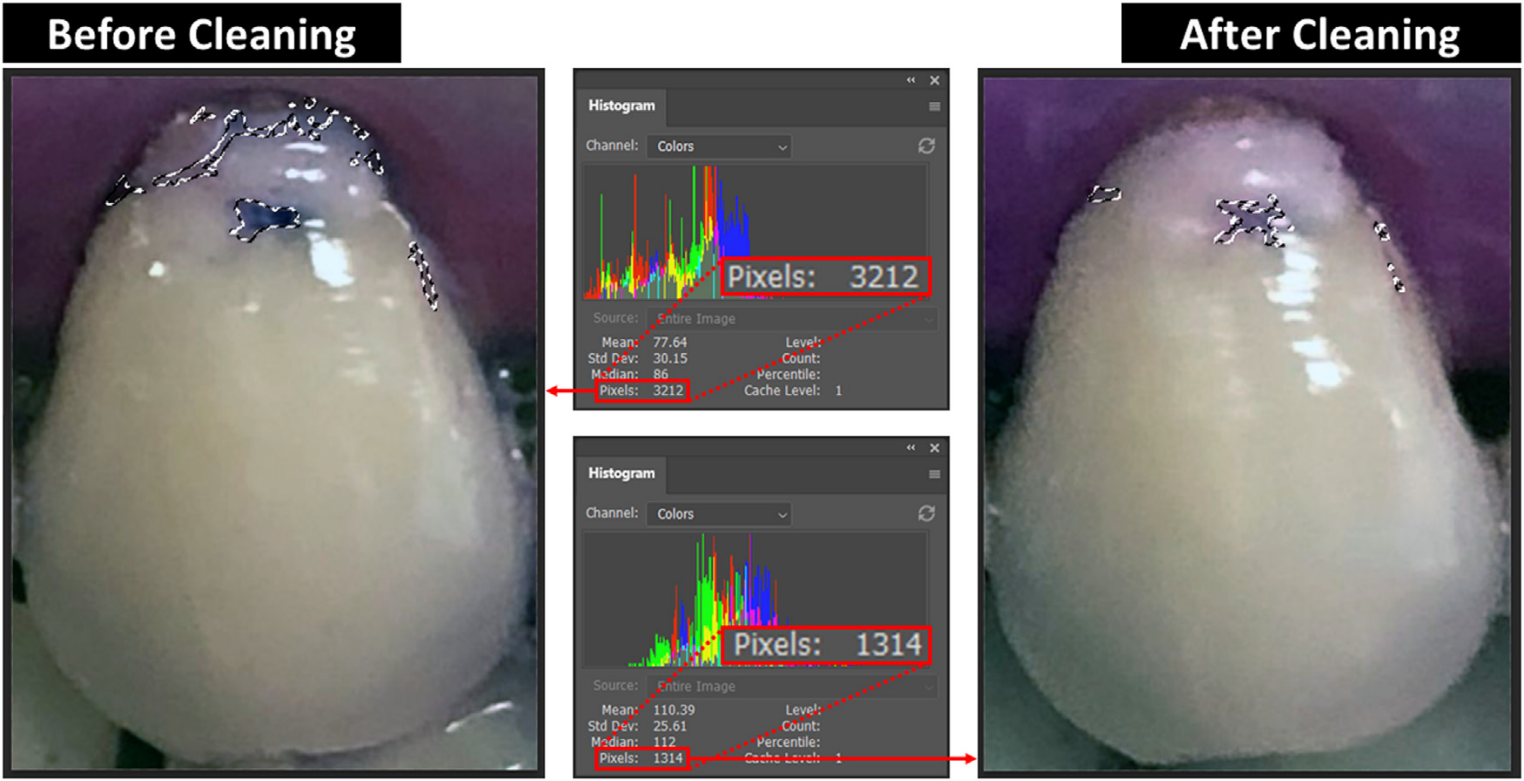
A demonstration of pixel-based plaque examination method.

As there are only 5 grades in TMQHPI, the sensitivity is low in plaque examination using TMQHPI. To improve the sensitivity and reduce the potential subjectivity of human plaque examination procedures, we have introduced a pixel-based plaque evaluation method on top of the traditional plaque index examination methods. All the evaluations were done in Photoshop 2021 software (Adobe). Teeth photos were first segmented into single tooth photos, and then dyed tooth photos before and after mouthguard cleaning were resized to exactly the same size (in pixels), so that the total pixels would be the same for the 2 photos before and after cleaning. To ensure the accuracy of tooth images to be measured, the camera was switched to manual mode with no automatic adjustments such as white balance, colour, contrast, or brightness. On the other hand, the tone, contrast, and colour were corrected in the Photoshop software. To measure the plaque-occupied area, the dyed area on tooth was selected by assigning a colour range to the software. All the pixels that lie in the selected colour range would be selected, and then the number of selected pixels can be shown accordingly (Figure 2).

The plaque removal rate of the mouthguard was calculated using the data from both TMQHPI and pixel-based method by Equations 1 and 2, respectively.

(1) $$\text{Plaque}\;\text{removal}\;\text{rate}{\;}_{\left(\text{TMQHPI}\right)}=\frac{\sum \left(\text{TMQHPI}\;before\;cleaning-\text{TMQHPI}\;after\;cleaning\right)}{\sum \text{TMQHPI}\;before\;cleaning}\times \;100\%$$

(2) $$\text{Plaque}\;\text{removal}\;\text{rate}{\;}_{\left(\text{pixel}-\text{based}\right)}=\frac{\sum \left(Dyed\;pixels\;before\;cleaning-Dyed\;pixels\;after\;cleaning\right)}{\sum Dyed\;pixels\;before\;cleaning}\times \;100\%$$

## Results

The results of plaque removal rate based on different sex and different tooth numbers are shown in the Table. The custom-made mouthguard can reduce the plaque attached on the teeth. The pixel-based calculating method was more sensitive to the dyed tooth occupied area. Whilst the traditional TMQHPI method only has 5 index grades, the amount and degree (ie, the shade of the dye) of plaque reduction within the same plaque index grade cannot be reflected properly, such that a higher plaque removal rate is obtained using a pixel-based method.

**Table:** Plaque removal rate based on different sex and tooth number.

		Plaque removal rate using TMQHPI	Plaque removal rate using pixel-based method
Sex	Male	5.68%	32.53%
	Female	8.36%	38.73%
	All participants	7.48%	36.35%
Tooth number	11	7.00%	36.16%
	12	6.70%	39.75%
	13	11.50%	31.21%
	31	6.60%	38.24%
	32	5.20%	31.68%
	33	7.60%	41.09%

TMQHPI, Turesky Modification of the Quigley-Hein Plaque Index.

## Discussion

The study demonstrated that the personalised 3D-printed micro-mist injection mouthguard can be effective in dental plaque removal on teeth and gingivae. The mouthguard uses micro-mist spraying to teeth and gingivae at high speed to remove dental plaque accumulations. As the micro-diameter water droplets in the micro-mist exhibit small size, light weight, and high kinetic energy it is able to remove plaque without causing dental hyperesthesia, pain, or injury.

Results (Table) revealed that female participants had a higher plaque removal rate using the mouthguard compared with male participants. This may be due to the existence of a higher amount of mature dental plaque amongst male participants (mature dental plaque is more difficult to clean compared with plaque in early stages). Ultimately, such a result could contribute to the difference in oral hygiene habits between male and female patients.^14^^,^^15^ There is no obvious relationship between tooth number and plaque removal effectiveness, as the Table indicates. This could be related to the design of the mouthguard: Multiple water and air nozzles are distributed evenly in the mouthguard, so the teeth can be cleaned thoroughly with no dead angles.

**Fig. 3:**
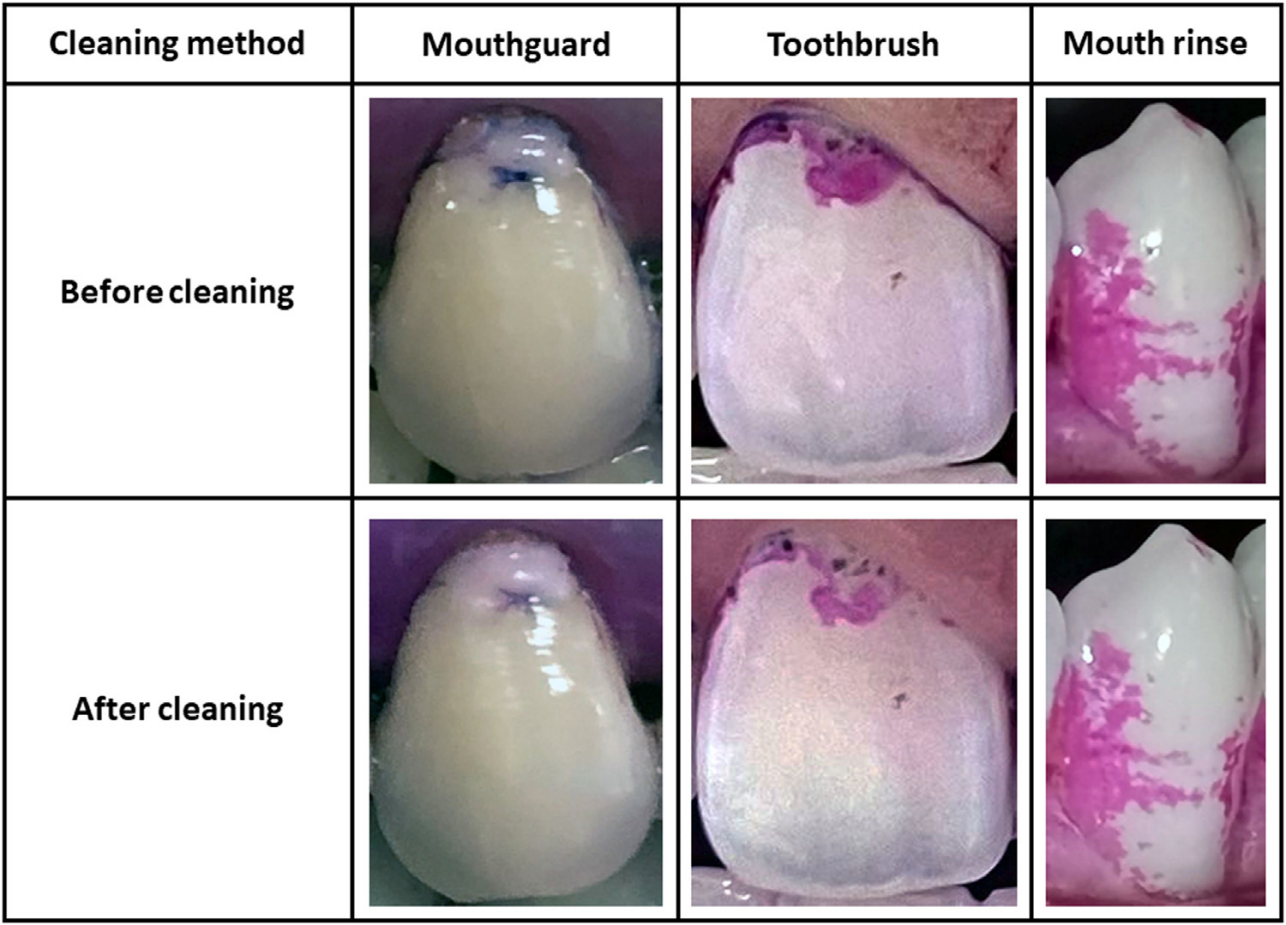
Images of tooth surface cleaned by different plaque removal method.

Comparing the plaque removal effectiveness of the mouthguard with other common methods, a manual toothbrush with water (normal brushing force, 15 seconds) can achieve a 27.78% (TMQHPI) or 72.70% (pixel-based) plaque removal rate, whilst rinsing with 15 mL mouth rinse for 30 seconds can achieve a 0.0% (TMQHPI) or 17.82% (pixel-based) plaque removal rate.^16^ Although the TMQHPI results indicated that the plaque removal rates were lower than 30% for all 3 cleaning methods, on image comparison (Fig. 3), it can be clearly seen that the dye becomes lighter after mouthguard or toothbrush cleaning, indicating that the amount of plaque becomes less after cleaning. The difference in results from TMQHPI and the pixel-based method can be due to the low sensitivity of the TMQHPI method.

Toothbrushing with toothpaste is considered an effective method for tooth cleaning, and it is one the universal tools for mechanical plaque removal. However, if abused orincorrectly used it may lead to tooth wear^17^ and/or gingival recession.^18^ In addition, the toothbrush can be easily contaminated if it is not properly stored and maintained, which may cause microbial growth on the toothbrush, their transfer to the oral cavity during brushing, and initiating periodontal disease.^19^ An oral irrigator (dental water jet, water flosser) is another cleaning device that utilises a water jet and airflow to clean dental plaque. This technology has been in use for 60 years,^20^ but it is not as frequently used by the public as the toothbrush. The oral irrigator has been shown to be efficacious in maintaining gingival health, such as reduction in periodontal pocket depth, bleeding, inflammation, and periodontal pathogens.^21^, ^22^, ^23^, ^24^ However, its effectiveness in reducing dental plaque varies. Husseini et al^25^ conducted a systematic review and found that oral irrigators do not have a beneficial effect in reducing visible plaque.

The mouthguard, on the other hand, uses mist generated by the air and water channel to clean the tooth without the device contacting the tooth directly—that is, by physical abrasion using toothbrush and toothpaste—so the risk of tooth wear and gingival recession is low compared with toothbrushing with toothpaste. The pressure of water and air channel of the mouthguard can be adjusted accordingly, thus avoiding unbalanced force during bruising using a traditional toothbrush, which may cause tooth wear and gingival recession.

Older adults and injured and disabled persons may not have complete flexibility in the arms and wrist movement to thoroughly clean their teeth with instruments such as toothbrushes, oral irrigators, and dental floss. The mouthguard device is especially suitable for such groups as they enabke tooth cleaning with no need to manually hold the cleaning instrument during the cleaning process. Instead, it can clean all the teeth simultaneously and obviate any blind niches that will be missed during manual brushing. Plus, the low volume of the irrigant used (5 mL to 10 mL for 10 seconds in a clinical trial) is unlikely to be a chocking hazard. Thus, the mouthguard device is a safe way to maintain users’ oral hygiene. It should be noted that the toothbrush is still considered the most effective way to remove dental plaque, whilst the mouthguard provides an alternative for oral self-care when toothbrushing is not feasible.

The pixel-based plaque examination method is a practical tool for assessing plaque formation with high sensitivity. However, because the method is based on intraoral photos, only anterior teeth can be captured and quantified clearly. Further work on this project includes antibacterial tests of 3D-printed materials and improving their antibacterial properties through material modification. It is worth investigating the effectiveness of mouthguard cleaning by older adults compared to conventional techniques, such as toothbrushing or oral irrigators, by caregivers. In addition, taking intraoral scanning for patients with autism spectrum disorder or attention deficit hyperactivity disorder may not be easy, due to their uncooperative behaviour, reluctance and anxiety about dental treatment. Thus, develop personalised mouthguards for them would be difficult. Developing a quick measurement methodology of plaque removal is essential to benefit a broader scope of potential users. Besides, a general model of the mouthguard with different sizes (i.e., small, medium, and large) can be batch produced for general public use.

## Conclusions

The personalised 3D-printed micro-mist injection mouthguard is proved to be efficacious in reducing dental plaque in anterior teeth, and the effectiveness lies between that of a toothbrush and that of mouth rinse. The mouthguard uses mist to clean the plaque instead of physical abrasion using toothbrush and toothpaste. The device can clean all the teeth simultaneously using a simple insertion procedure while older adults and especially disabled people can benefit from this device with minimal, if any, manual brushing.

## Acknowledgements

Hao Ding and Meng Zhang contributed equally to this article. The authors would like to acknowledge the funding support from Innovation and Technology Fund for Better Living (FBL), Innovation and Technology Commission, Hong Kong SAR Government (ITB/FBL/6020/18/S). We also acknowledge the secretarial support from Miss Bobo Kong, volunteering BDS students who were participated as helpers, and the older adult participants in this clinical trial.

## Conflict of interest

None disclosed.
